# Combined HER3-EGFR score in triple-negative breast cancer provides prognostic and predictive significance superior to individual biomarkers

**DOI:** 10.1038/s41598-020-59514-1

**Published:** 2020-02-20

**Authors:** Angela Ogden, Shristi Bhattarai, Bikram Sahoo, Nigel P. Mongan, Mansour Alsaleem, Andrew R. Green, Mohammed Aleskandarany, Ian O. Ellis, Sonal Pattni, Xiaoxian (Bill) Li, Carlos S. Moreno, Uma Krishnamurti, Emiel A. Janssen, Kristin Jonsdottir, Emad Rakha, Padmashree Rida, Ritu Aneja

**Affiliations:** 1 Department of Biology, Georgia State University, Atlanta, GA USA; 2 Faculty of Medicine and Health Science, School of Veterinary Medicine and Science, University of Nottingham, Nottingham, LE12 5RD UK; 3 Department of Pharmacology, Weill Cornell Medicine, 1300 York Ave., NY USA; 4 Nottingham Breast Cancer Research Centre, Division of Cancer and Stem Cells, School of Medicine, University of Nottingham Biodiscovery Institute, University Park, Nottingham, NG7 2RD UK; 5 Department of Pathology and Laboratory Medicine, Emory University School of Medicine, Atlanta, GA USA; 6 Department of Pathology, Stavanger University Hospital, Stavanger, Norway; 7 Department of Chemistry, Bioscience and Environmental Engineering, University of Stavanger, 4036 Stavanger, Norway; 8 Novazoi Theranostics, Inc., Rolling Hills Estates, CA USA

**Keywords:** Gene expression analysis, Breast cancer

## Abstract

Epidermal growth factor receptor (EGFR) and human epidermal growth factor receptor 3 (HER3) have been investigated as triple-negative breast cancer (TNBC) biomarkers. Reduced EGFR levels can be compensated by increases in HER3; thus, assaying EGFR and HER3 together may improve prognostic value. In a multi-institutional cohort of 510 TNBC patients, we analyzed the impact of HER3, EGFR, or combined HER3-EGFR protein expression in pre-treatment samples on breast cancer-specific and distant metastasis-free survival (BCSS and DMFS, respectively). A subset of 60 TNBC samples were RNA-sequenced using massive parallel sequencing. The combined HER3-EGFR score outperformed individual HER3 and EGFR scores, with high HER3-EGFR score independently predicting worse BCSS (Hazard Ratio [HR] = 2.30, p = 0.006) and DMFS (HR = 1.78, p = 0.041, respectively). TNBCs with high HER3-EGFR scores exhibited significantly suppressed ATM signaling and differential expression of a network predicted to be controlled by low TXN activity, resulting in activation of EGFR, PARP1, and caspases and inhibition of p53 and NFκB. Nuclear PARP1 protein levels were higher in HER3-EGFR-high TNBCs based on immunohistochemistry (p = 0.036). Assessing HER3 and EGFR protein expression in combination may identify which adjuvant chemotherapy-treated TNBC patients have a higher risk of treatment resistance and may benefit from a dual HER3-EGFR inhibitor and a PARP1 inhibitor.

## Introduction

Accruing evidence suggests that ErbB family members may be useful biomarkers of aggressive disease course and potential drug targets in breast cancer, including triple-negative breast cancer (TNBC), which is defined by the biomarkers/drug targets it lacks, including amplification of ErbB family member HER2. The human epidermal growth factor receptor (EGFR) family consists of four tyrosine kinase receptors (EGFR/HER1, HER2, HER3, and HER4) that stimulate growth signaling pathways involved in cell proliferation, growth, survival, and differentiation. EGFR is overexpressed in at least 50% of TNBCs, which is notably higher than in other BC subtypes depending on the cohort characteristics and staining/scoring protocols^1^. High EGFR copy number, immunoreactivity and membrane expression have been shown to be independent prognostic indicators of poorer overall survival (OS) and disease-free survival (DFS) in TNBC, suggesting EGFR to be a potentially targetable and risk-predictive biomarker in TNBC^2–5^. HER3 is the only receptor in the family that is catalytically inactive and requires dimerization with other members in order to be activated^6^. HER3 overexpression has been reported in approximately 20–30% of invasive breast carcinomas^7^. However, the value of HER3 and EGFR as prognostic biomarkers in TNBC remains uncertain. One study found that HER3-positivity was an independent predictor of poor disease-free survival in breast cancer after adjusting for breast cancer subtype and other potential confounders, whereas EGFR-positivity did not independently predict disease-free survival^8^. Reduced expression of EGFR members can be compensated by increased expression of HER3, which promotes resistance to EGFR inhibitors^8,9^. Since EGFR expression varies with that of HER3, these proteins may need to be considered jointly to be useful biomarkers. Thus, the aims of the present study were to (i) determine whether combined HER3-EGFR protein expression in pre-treatment resection specimens could independently predict survival in TNBC patients, (ii) compare the prognostic value of HER3-EGFR to individual HER3 and EGFR protein expression, and (iii) identify potential targets for therapy based on stratification by combined HER3-EGFR protein expression in RNA-sequenced pre-treatment TNBCs.

## Materials and Methods

### Ethics approval and consent to participate

Nottingham cohort: This study was approved by the Nottingham Research Ethics Committee 2 under the title “Development of a molecular genetic classification of breast cancer,” as well as the Institutional Review Boards (IRB) of the respective institutions. All samples from Nottingham used in this study were pseudo-anonymized and collected prior to 2006 and therefore under the Human Tissue Act informed patient consent was not needed. Release of data was also pseudo-anonymized as per Human Tissue Act regulations. Stavanger cohort: The use of the Stavanger material was approved by the Regional Ethical Committee (REC) (2010/1241). REC also approved that informed patient consent was not needed. All insights in a patient’s journal were monitored electronically, and all, except the treating physician, are required to state the reason why they read a patient’s journal. This log is always available for all patients. All data and biological material provided were pseudo-anonymized. Emory cohort All samples from the Emory cohort were archival tissue for which informed consent was not required as approved by the Emory IRB. All data and biological material provided were pseudo-anonymized.

### Datasets, specimens, immunohistochemistry, and scoring

TNBCs were identified as lacking ER and PR expression and lacking HER2 over-expression and/or gene amplification (i.e., ER−/PR−/HER2−). Datasets were compiled from Nottingham City Hospital, Nottingham UK (n = 302); Stavanger University Hospital, Stavanger, Norway (n = 104); and Emory University Hospital, Atlanta, GA, US (n = 104), all consisting of TNBC patients with primary operable invasive disease. Biomarkers were evaluated prior to any treatment. For the Nottingham and Emory cohorts, specimens comprised tissue microarrays, whereas for the Stavanger cohort, specimens comprised full-face tissue sections. All slides were centrally reviewed.

### Immunohistochemistry and scoring

Nottingham specimens were from patients belonging to the well-characterized Nottingham Tenovus Primary Breast Carcinoma Series^10^, for which HER3 and EGFR scores from tissue microarrays had been previously determined as described by Abd El-Rehim *et al*.^11^. Tissue microarrays (Emory cohort) or full-face formalin-fixed paraffin-embedded tissue slides (Stavanger cohort) were deparaffinized and then rehydrated in serial ethanol baths (100%, 90%, 75% and 50%). For HER3, antigen retrieval was performed using the Dako Target Retrieval Solution at pH 9 in a 90 °C water bath for 35 min. Then, slides were incubated with monoclonal mouse anti-human HER3 antibody (DAK-H3-IC) at 1:25 for 30 min. For EGFR, antigen retrieval was performed using Proteinase K (Agilent, S3020) for 6 min on a hot rack. Then, samples were incubated with mouse anti-human EGFR antibody (Life Technologies, S3020) at 1:20 for 30 min. Finally, the Dako EnVision™ + Dual Link System-HRP system (K4065) was used for both anti-HER3 and anti-EGFR-labeled slides according to the manufacturer’s instructions, followed by counterstaining with hematoxylin. Stavanger and Emory specimens were scored as in the Nottingham series (H-scores) by the same experienced breast pathologist (for HER3) or independently by two experienced breast pathologists (for EGFR), with the average score used in downstream analysis. Combined HER3-EGFR protein expression was computed as the sum of the individual HER3 and EGFR H-scores. Descriptive statistics can be found in Table S1.

### Exploring differences in biomarker scores by cohort

Mean HER3, EGFR, and HER3-EGFR protein expression varied by cohort (ANOVA p < 10^−6^ for all); specifically, according to Tamhane’s T2 post-hoc test, the means were higher in the Nottingham cohort than the other cohorts (p < 0.05 for all); mean HER3 was higher in the Stavanger than Emory cohort (p < 10^−6^); mean EGFR was higher in the Emory than Stavanger cohort (p = 1 × 10^−6^); and mean HER3-EGFR did not differ between Stavanger and Emory cohorts (p = 0.40) (Tables S2 and S3). Variation in HER3, EGFR, and HER3-EGFR was explored via categorical regression with optimal scaling using the CATREG procedure. Specifically, HER3, EGFR, HER3-EGFR, age at diagnosis were scaled numerically and discretized by multiplying; Nottingham grade and AJCC stage were scaled ordinally and discretized by ranking; adjuvant chemotherapy (yes/no) was scaled nominally and discretized by grouping (uniform). The initial configuration was set as multiple systemic starts. All other settings were default. Variation in expression of HER3, EGFR, and HER3-EGFR was primarily explained by the cohort rather than other patient or clinicopathologic variables (Table S4).

### RNA sequencing

RNA-sequencing was performed on 60 formalin-fixed paraffin-embedded TNBC pre-treatment samples for which HER3 and EGFR scores were available (full-face slides), which were taken from patients in the Nottingham cohort who were eventually treated with adjuvant CMF chemotherapy. Cases were selected based on which had sufficient material remaining for RNA-seq. RNA-seq data were deposited in ArrayExpress (accession: E-MTAB-6729), where detailed methods can be found. In brief, samples were processed by the Emory Integrated Genomics Core using the Mag-Bind XP FFPE RNA isolation kit (Omega), KingFisher Flex magnetic particle separator (ThermoFisher), TruSeq RNA Access library kit (Illumina), Agilent 2200 TapeStation (Agilent Technologies), the QuantiFluor dsDNA System (Promega), and the Agilent High Sensitivity D1000 ScreenTape on the Agilent 2200 Tapestation (Agilent Technologies), and the HiSeq2500 (Illumina, Inc.) per the manufacturers’ instructions.

### Analysis of RNA-seq data

The Salmon *index* command was used for transcriptome index was building on GRCh38.P10, after which alignment-free transcript abundance was quantified^12^. Gene-level abundance was estimated using tximport^13^. Batch effects were removed using the SVA package^14^. The DESeq2 approach^15^ was used to determine differential expression with and without adjusting for age at diagnosis and AJCC stage (Tables S5 and S6). Ingenuity Pathway Analysis (IPA) was used to identify differentially regulated canonical pathways and causal networks based on 1,378 transcripts (out of 35,590) differentially expressed at the FDR q < 0.05 level in age- and stage-adjusted differential expression analysis.

### IPA causal network analysis

Causal network analysis was performed in IPA with the settings adjusted to include only genes, RNAs, and proteins (e.g., rather than drugs or functions). The expression log_2_ ratio used to calculate directionality (Z-score). The list of predicted causal networks was filtered to include only hits with significant z-scores (Z-score > 2) without apparent bias. This is to say, we excluded regulators with |µ| < 0.25, where “bias” or $\mu ={\mu }_{data}+{\mu }_{regulator}$; ${N}_{up}$ and ${N}_{down}$ are the numbers of up and down-regulated genes, respectively; ${N}_{activating}$ and ${N}_{inhibiting}$ are genes to which the regulator is connected through activating and inhibiting edges; and $${\begin{array}{c}\mu \end{array}}_{data}=\frac{{N}_{up}-{N}_{down}}{{N}_{up}+{N}_{down}}$$; ${\mu }_{regulator}=\frac{{N}_{activating}-{N}_{inhibiting}}{{N}_{activating}+{N}_{inhibiting}}$, all per the manufacturer’s white book on Ingenuity Upstream Regulator Analysis in IPA®.

### Survival analyses

Survival outcomes were defined as the time from the date of diagnosis to death from breast cancer (BCSS) or distant metastasis (DMFS). Continuous HER3, EGFR, and HER3-EGFR protein expression levels were converted to categorical variables based on the medians of the entire multi-institutional cohort (95, 0, and 121, respectively). Satisfaction of the proportional hazards assumption was tested by entering time-dependent covariates in univariate Cox models and verifying p > 0.05 for all of them. The impact of categorical HER3, EGFR, and HER3-EGFR expression in pre-treatment resection samples on BCSS and DMFS was testing in subgroups based on adjuvant chemotherapy status (none vs. treatment received) using univariate and multivariate (age and stage-adjusted) Cox regression. Survival analyses were stratified by cohort (i.e., different baseline survival functions were computed for each stratum) to adjust for the potentially confounding influence of cohort differences (due to correlation with protein expression levels and independent prognostic value) without estimating their effects on survival. Results of survival analyses were considered significant if p < 0.05. Analyses were conducted using IBM SPSS Statistics v. 21.

### Analysis of PARP1 by IHC

Nuclear non-cleaved PARP1 H-scores in pre-treatment FFPE resection samples were available from TNBC patients in the Nottingham cohort who ultimately received adjuvant chemotherapy, the staining and scoring of which have previously been described by Green *et al*.^16^. PARP1 H-scores were compared between low/high HER3-EGFR groups by a two-tailed Mann Whitney U test (n = 38 and n = 93, respectively) using SPSS.

## Results

**Figure 1:**
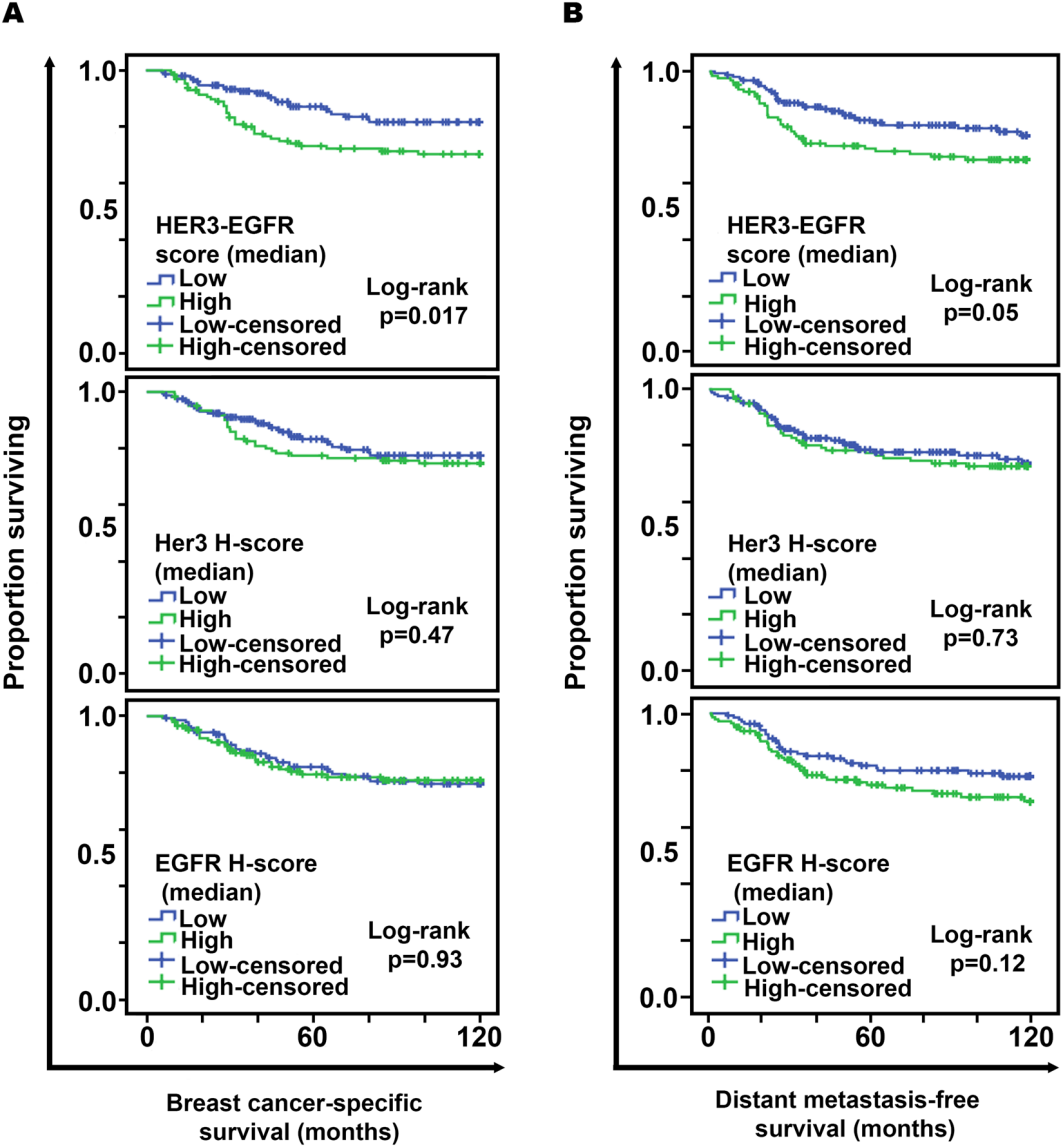
Product-limit survival estimates over 10 years of observation of adjuvant chemotherapy-treated TNBC patients based on combined HER3-EGFR (top), HER3 (middle), and EGFR (bottom) protein expression. (**A**) Breast cancer-specific survival. (**B**) Distant metastasis-free survival.

In Kaplan-Meier analysis, high combined HER3-EGFR protein expression in pre-treatment resection specimens from TNBC patients conferred worse 10-year BCSS (83.1% vs 69.2%, log-rank p = 0.017) and DMFS (80.8% vs 70.4%, log-rank p = 0.05) after adjuvant chemotherapy. Moreover, patients with low HER-EGFR has a better prognosis compared to other groups (Fig. 1). In univariate Cox models, high HER3-EGFR was associated with 2.50-fold increased risk of dying from breast cancer after adjuvant chemotherapy (p = 0.003) but not when adjuvant chemotherapy was not administered (Table 1). By contrast, HER3 and EGFR individually had no effect on BCSS regardless of adjuvant chemotherapy status; similar results were obtained in analyses of DMFS. High HER3-EGFR was associated with 1.95-fold increased risk of distant metastasis after adjuvant chemotherapy (p = 0.022) but not when adjuvant chemotherapy was not administered (Table 1). Neither HER3 nor EGFR significantly impacted DMFS regardless of adjuvant chemotherapy status.

**Table 1:** Risk of worse breast cancer-specific or distant metastasis-free survival conferred by high protein levels HER3, EGFR, or HER3-EGFR protein expression in univariate Cox models stratified by hospital.

Biomarker	Chemo	Breast cancer-specific survival				Dstant metastasis-free survival			
				95% CI for HR				95% CI for HR	
		p-value	HR	Lower	Upper	p-value	HR	Lower	Upper
HER3	No	1.00	1.00	0.52	1.92	0.81	0.93	0.51	1.69
	Yes	0.41	1.31	0.69	2.47	0.58	1.19	0.63	2.25
EGFR	No	0.91	1.03	0.58	1.84	0.80	1.08	0.62	1.86
	Yes	0.63	1.13	0.69	1.84	0.10	1.48	0.92	2.38
HER3-EGFR	No	0.97	1.01	0.55	1.84	0.89	0.96	0.55	1.68
	Yes	**0**.**003**	2.50	1.38	4.53	**0**.**022**	1.95	1.10	3.43

HR = Hazard Ratio, CI = Confidence Interval.

In multivariate analysis, high HER3-EGFR remained a significant predictor of worse BCSS after adjuvant chemotherapy (Confidence Interval [CI] = 1.26–4.20, hazard ratio [HR] = 2.30, p = 0.006) but not when patients did not receive adjuvant chemotherapy (Table 2). Similarly, high HER3-EGFR independently predicted worse DMFS after adjuvant chemotherapy (Confidence Interval [CI] = 1.02–3.11, HR = 1.78, p = 0.041) but not when patients did not receive adjuvant chemotherapy (Table 2). Neither HER3 nor EGFR significantly impacted BCSS or DMFS regardless of adjuvant chemotherapy status.

**Table 2:** Risk of worse breast cancer-specific or distant metastasis-free survival conferred by high protein levels of HER3, EGFR, or HER3-EGFR in age- and stage-adjusted Cox models stratified by hospital.

Model	Covariate	Chemo	Breast cancer-specific survival				Distant metastasis-free survival			
			p-value	HR	95% CI for HR		p-value	HR	95% CI for HR	
					Lower	Upper			Lower	Upper
HER3	HER3	No	0.58	1.21	0.62	2.36	0.77	1.10	0.59	2.06
	Age		0.96	1.00	0.97	1.03	0.99	1.00	0.97	1.03
	Stage		**<0**.**001**				**<0**.**001**			
	II vs. I		0.51	1.24	0.65	2.38	0.27	1.42	0.76	2.63
	III vs. I		**<0**.**001**	6.63	2.96	14.85	**<0**.**001**	6.09	2.67	13.92
	HER3	Yes	0.57	1.21	0.63	2.34	0.72	1.12	0.59	2.11
	Age		0.10	1.02	1.00	1.05	1.00	1.00	0.98	1.02
	Stage		**0**.**010**				**0**.**020**			
	II vs. I		0.79	1.10	0.57	2.12	0.97	1.01	0.56	1.85
	III vs. I		**0**.**014**	2.58	1.21	5.49	**0**.**022**	2.36	1.13	4.93
EGFR	EGFR	No	0.75	0.91	0.51	1.63	0.86	0.95	0.54	1.68
	Age		0.98	1.00	0.97	1.03	0.98	1.00	0.97	1.03
	Stage		**<0**.**001**				**<0**.**001**			
	II vs. I		0.56	1.21	0.64	2.30	0.28	1.40	0.76	2.59
	III vs. I		**<0**.**001**	6.42	2.89	14.27	**<0**.**001**	6.02	2.64	13.75
	EGFR	Yes	0.67	1.11	0.68	1.81	0.18	1.39	0.86	2.23
	Age		0.09	1.02	1.00	1.05	0.89	1.00	0.98	1.03
	Stage		**0**.**008**				**0**.**024**			
	II vs. I		0.81	1.09	0.56	2.10	0.98	1.01	0.55	1.84
	III vs. I		**0**.**013**	2.59	1.22	5.51	**0**.**027**	2.30	1.10	4.81
HER3-EGFR	HER3-EGFR	No	0.85	0.94	0.51	1.74	0.78	0.92	0.51	1.65
	Age		0.98	1.00	0.97	1.03	0.98	1.00	0.97	1.03
	Stage		**<0**.**001**				**<0**.**001**			
	II vs. I		0.58	1.20	0.63	2.29	0.30	1.39	0.75	2.56
	III vs. I		**<0**.**001**	6.33	2.88	13.96	**<0**.**001**	5.99	2.66	13.48
	HER3-EGFR	Yes	**0**.**006**	2.30	1.26	4.20	**0**.**041**	1.78	1.02	3.11
	Age		0.13	1.02	0.99	1.05	0.83	1.00	0.98	1.03
	Stage		**0**.**016**				**0**.**007**			
	II vs. I		0.73	1.12	0.58	2.17	0.83	1.07	0.58	1.95
	III vs. I		**0**.**018**	2.49	1.17	5.31	**0**.**009**	2.61	1.28	5.32

HR = Hazard Ratio, CI = Confidence Interval.

**Figure 2:**
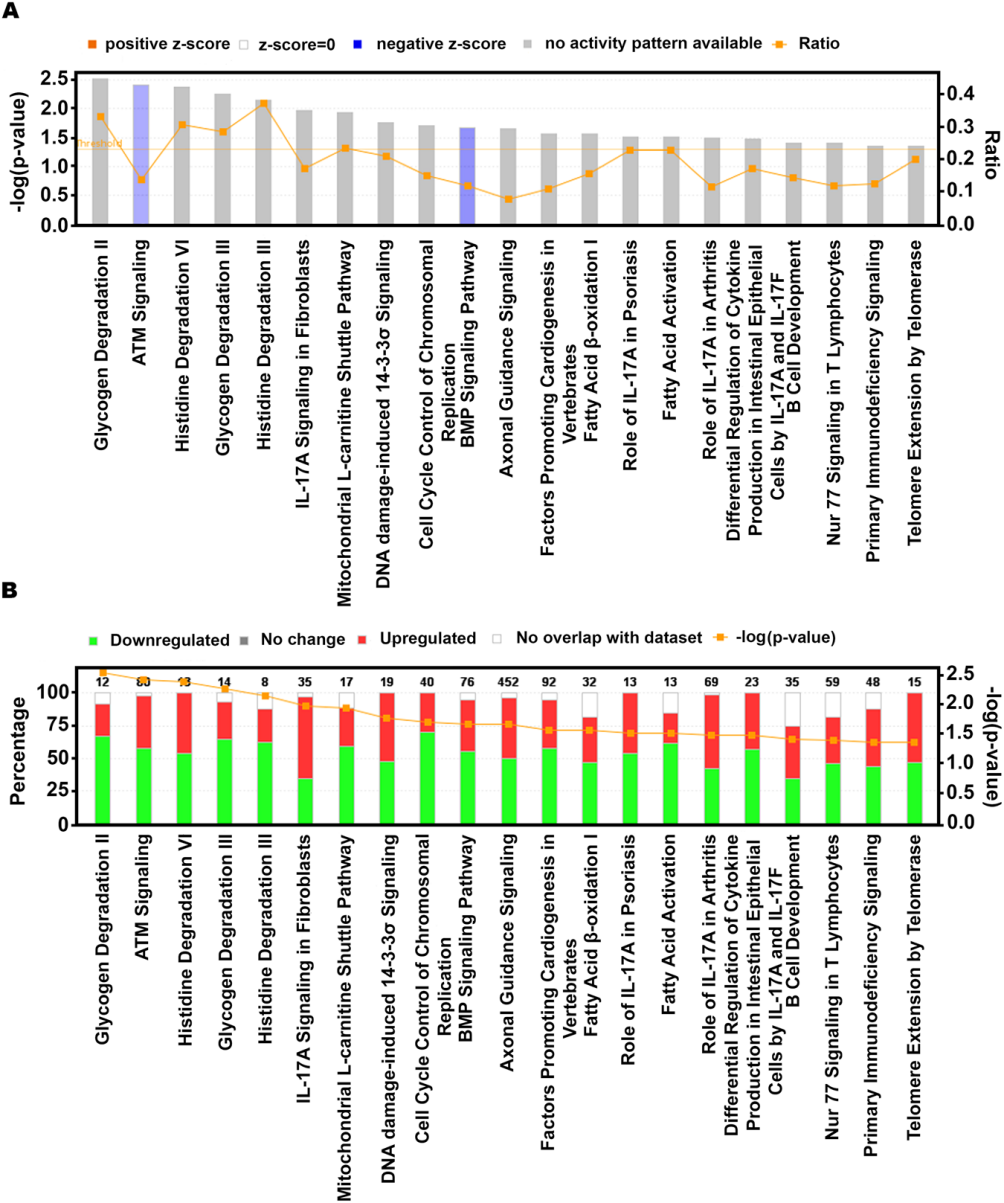
Differentially regulated canonical pathways from Ingenuity Pathway Analysis. (**A**) The significance of each pathway (bar graphs; left y-axis), the log_2_ fold-change (line graph; right y-axis), and z-score/activity pattern (bar color). (**B**) Stacked bar chart depicting the percentage (bar graph; left y-axis) of pathway components up-, down-, or not differentially expressed (bar color), with the number of pathway components given over each respective bar, and the significance of the differential regulation (line graph; right y-axis). Only transcripts with q < 0.05 were entered in the analysis; all pathways depicted exhibit p < 0.05.

**Figure 3:**
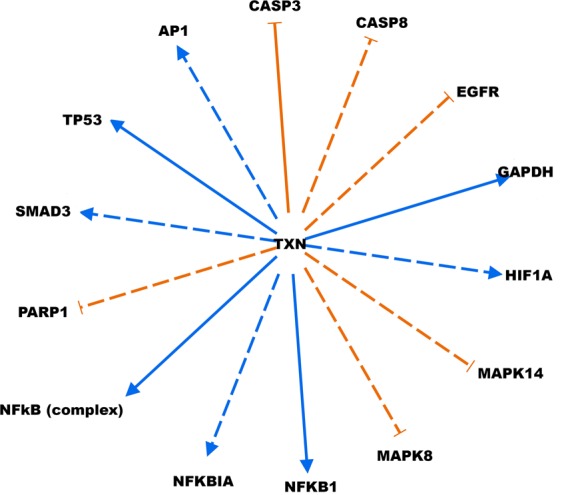
Top causal network explaining observed gene expression differences. Master regulator TXN is at the hub of the network, controlling upstream regulators of observed gene expression difference, including EGFR. Solid line: direct interaction; broken line: indirect interaction. Orange: Predicted activation; Blue: predicted inhibition. Causal network analysis was conducted using Ingenuity Pathway Analysis tools.

Next, we analyzed differences in pathways, upstream regulators, and networks between HER3-EGFR groups in TNBC patients who received adjuvant chemotherapy to reveal potentially actionable biology in the HER3-EGFR-high, poor-prognosis group. RNA-seq data for a subset of 60 TNBCs in the Nottingham cohort were analyzed (21 with low HER3-EGFR, 39 with high HER3-EGFR). Neither HER3 nor EGFR was differentially regulated based on HER3-EGFR (q = 0.97 and q = 0.95, respectively; Table S5), reflecting a disconnect between transcript levels and expression of these proteins at the plasma membrane. After adjusting for age and stage in differential expression analysis (Table S6), the top differentially regulated canonical pathway was ATM signaling, which was lower in the HER3-EGFR-high group (z = −1.27, p = 0.004; Fig. 2). Aligned with this finding, DNA damage-induced 14-3-3σ signaling was significantly deregulated by HER3-EGFR group, with about half of the pathway genes downregulated and half upregulated (p = 0.017; Fig. 2). The predicted top master regulator whose activity could explain observed transcriptional differences was the enzyme thioredoxin (TXN) (Table 3). Intriguingly, this causal network includes EGFR, DNA damage sensor PARP1, and caspases 3 and 8 (all predicted to be activated), and p53 and NFκB (predicted to be inhibited) (Fig. 3 and Table 3). Consistent with these results, the top molecular/cellular function was Cell Death and Survival, with significant activation of genes involved in death/apoptosis of cardiac and brain cells (activation z scores = 0.24 and 0.50, p = 0.014 and 0.016, respectively). Thus, TNBCs exhibiting high HER3-EGFR protein expression may be susceptible to inhibitors of the DNA damage response. Of note, the second-top hit in causal network analysis was reticulon-1 (RTN1), predicted to be activated and a regulator of TP53, TERT, and various MAPKs.

**Table 3:** Predicted causal networks explaining observed gene expression differences based on combined HER3-EGFR protein expression.

Master Regulator	Log_2_FC	Participating regulators	Predicted Activation State	Activation z-score	p-value of overlap	corrected p-value
TXN	−1.93	Ap1, CASP3, CASP8, EGFR, GAPDH, HIF1A, MAPK14, MAPK8, NFkB (complex), NFKB1, NFKBIA, P38 MAPK, PARP1, SMAD3, TP53, TXN	Inhibited	−2.18	1.64E-02	8.80E-03
RTN1	2.05	BCL2L1, BID, CASP3, CASP8, F3, GSK3B, ITPR1, Jnk, Mapk, MAPK8, NFkB (complex), P38 MAPK, Pdi, RTN1, TERT, TP53, UGCG	Activated	2.54	1.77E-02	8.50E-03
TPSD1	−1.33	MAPK1, MAPK14, MAPK3, MAPK8, MAPK9, RARRES2, TPSD1	Inhibited	−2.11	1.82E-02	6.50E-03
HDAC1	−1.28	AR, E2F3, E2F4, EGFR, HDAC1, KDM1A, LEF1, PPARG, RB1, Smad2/3-Smad4, SMAD7, TP53	Activated	3.28	3.56E-02	2.73E-02

Finally, we compared uncleaved nuclear PARP1 H-scores between HER3-EGFR groups and found that PARP1 was significantly higher in HER3-EGFR TNBCs from patients who ultimately received adjuvant chemotherapy (p = 0.036; mean rank = 40.24 vs. 53.39 in HER3-EGFR-low and high cases, respectively), consistent with our findings from IPA causal network analysis.

## Discussion

Our finding that combined HER3-EGFR protein expression, but not individual HER3 or EGFR protein expression, independently predicts worse BCSS and DMFS following adjuvant chemotherapy for TNBC suggests that HER3 and EGFR should be considered jointly since their expression has been empirically demonstrated to be linked, with decreased expression in one protein inducing compensatory upregulation of the other and thereby promoting resistance to targeted therapies^8,9^. We discovered that a transcriptional network in the HER3-EGFR-high group may be controlled by master regulator TXN (predicted to be inhibited), which may cause activation of PARP1, EGFR, and caspases 3 and 8, while also inhibiting p53, NFκB, AP1, SMAD3, and HIF1A, proteins that are all involved in regulating apoptosis, which may be abnormal in TNBCs with high HER3-EGFR protein expression. Intriguingly, TXN-negative breast cancers are more responsive to docetaxel^17^. Thus, regimens including docetaxel may be effective for HER3-EGFR-high TNBCs. Furthermore, we also identified the potential master transcriptional regulator RTN1, a protein that promotes endoplasmic reticulum-mediated apoptosis and is implicated in neurodegenerative diseases and cancer^18^. Thus, RTN1 may also represent a novel therapeutic target for TNBCs with high HER3-EGFR protein expression.

Limitations of the present study include its retrospective nature and differences in tissue fixation and immunohistochemistry between cohorts. However, strengths include the relatively large sample size, multivariate analysis that adjusted for possible confounders including cohort, and analysis of RNA-sequencing data. This work lays the foundation for *in vitro* and *in vivo* studies of combinatorial regimens including dual HER3/EGFR inhibitors, PARP1 inhibitors, and docetaxel-based chemotherapy in TNBCs exhibiting high combined HER3-EGFR protein expression. Importantly, PARP inhibitors have thus far not shown promise in unselected TNBC patients^19^. Therefore, a lack of technically feasible and cost-effective biomarkers to guide selection of TNBC patients for anti-PARP therapy is a critical barrier to progress in the field, which this study may help to address. Our results justify a retrospective analysis of HER3-EGFR in clinical trials or could be the basis for translational sub-projects in upcoming studies for patients with TNBC. Altogether, this work highlights the clinical value of assessing protein expression of HER3 and EGFR in combination which may potentially guide the selection of targeted drugs (dual HER3-EGFR and PARP1 inhibitors) and cytotoxic agents for TNBC patients with poor prognosis after adjuvant chemotherapy.

## Supplementary information

Supplementary file.

Supplementary Table5.

Supplementary Table6.

## Data Availability

The RNAseq and clinical data are freely available on ArrayExpress (accession: E-MTAB-6729).
