# Supplementary material for: Combined HER3-EGFR score in triple-negative breast cancer provides prognostic and predictive significance superior to individual biomarkers
Source: Sci Rep. 2020 Feb 20;10:3009. doi: 10.1038/s41598-020-59514-1 (PMC7033213; doi:10.1038/s41598-020-59514-1)
Supplement: Supplementary file 1 — Supplementary file. [file 41598_2020_59514_MOESM1_ESM.pdf]

Combined HER3-EGFR score in triple-negative breast cancer provides prognostic and predictive significance superior to individual biomarkers

Angela Ogden[1], Shristi Bhattarai[1], Bikram Sahoo[1], Nigel P. Mongan[2,3], Mansour Alsaleem[4], Andrew R. Green[4], Mohammed Aleskandarany[4], Ian O. Ellis[4], Sonal Pattni[5], Xiaoxian (Bill) Li[5], Carlos S. Moreno[5], Uma Krishnamurti[5], Emiel A. Janssen[6,7], Kristin Jonsdottir[6], Emad Rakha[2], Padmashree Rida[1,8*], Ritu Aneja[1*]

[1]Department of Biology, Georgia State University, Atlanta, GA, USA

[2]Faculty of Medicine and Health Science, School of Veterinary Medicine and Science, University of Nottingham, LE12 5RD, UK

[3]Department of Pharmacology, Weill Cornell Medicine, 1300 York Ave., NY, USA

[4]Nottingham Breast Cancer Research Centre, Division of Cancer and Stem Cells, School of Medicine, University of Nottingham, Nottingham NG5 1PB, UK

[5]Department of Pathology and Laboratory Medicine, Emory University School of Medicine, Atlanta, GA, USA

[6]Department of Pathology, Stavanger University Hospital, Stavanger, Norway

[7]Department of Mathematics and Natural Sciences, University of Stavanger, 4036 Stavanger, Norway

[8]Novazoi Theranostics, Inc., Rolling Hills Estates, CA, USA

***Corresponding Authors**

Ritu Aneja, Department of Biology, Georgia State University, Atlanta, GA, 30303; raneja@gsu.edu

Padmashree C. G. Rida, Department of Biology, Georgia State University, Atlanta, GA 30303; Novazoi Theranostics, Inc., Rolling Hills Estates, CA, 90274; cgp_rida@yahoo.com

Supplementary Material

Table S1. Clinicopathologic data of the study cohorts for the Nottingham, Stavanger, and Emory datasets.

Variable	Statistic (continuous variables) or level (categorical variables)	Hospital		
		Nottingham	Stavanger	Emory
HER3 H-score	Median	146	50	5
	Mean	146	53	20
	Standard Deviation	75	46	38
	Minimum	0	0	0
	Maximum	300	180	185
	Missing	0	0	0
EGFR H-score	Median	0	0	9
	Mean	36	12	61
	Standard Deviation	62	38	86
	Minimum	0	0	0
	Maximum	300	300	300
	Missing	0	0	0
Combined HER3-EGFR	Median	170	60	33
	Mean	182	64	80
	Standard Deviation	106	65	93
	Minimum	0	0	0
	Maximum	600	430	330
	Missing	0	0	0
Age at diagnosis (years)	Median	50	52	56
	Mean	50	54	57
	Standard Deviation	11	14	12
	Minimum	27	22	23
	Maximum	71	85	85
	Missing	3	0	0
AJCC stage	I	95	30	40
	II	171	61	50
	III	35	11	14
	Missing	1	2	0

Chemotherapy	No	147	0	13
	Yes	131	80	77
	Missing	24	24	14

Table S2. One-way ANOVA of HER3, EGFR, and HER3-EGFR by cohort.

Biomarker	Comparison	Sum of Squares	Mean Square	F	p
HER3	Between Groups	1536298.75	768149.37	189.22	4.11E-62
	Within Groups	2058172.50	4059.51		
	Total	3594471.25			
EGFR	Between Groups	123875.80	61937.90	15.25	3.71E-07
	Within Groups	2059527.20	4062.18		
	Total	2183403.00			
HER3-EGFR	Between Groups	1495972.41	747986.20	80.73	3.64E-31
	Within Groups	4697245.93	9264.78		
	Total	6193218.34			

Table S3. Tamhane's T2 post-hoc testing to detect for which cohorts mean HER3, EGFR, and HER3-EGFR differ.

Dependent Variable			Mean Difference (I-J)	Std. Error	p	95% Confidence Interval	
						Lower Bound	Upper Bound
HER3	Nottingham	Norway	93.10	6.22	0.00E+00	78.15	108.05
		Emory	126.11	5.70	0.00E+00	112.44	139.77
	Stavanger	Nottingham	-93.10	6.22	0.00E+00	-108.05	-78.15
		Emory	33.01	5.82	1.49E-07	18.99	47.02
	Emory	Nottingham	-126.11	5.70	0.00E+00	-139.77	-112.44
		Norway	-33.01	5.82	1.49E-07	-47.02	-18.99
EGFR	Nottingham	Norway	24.53	5.16	9.43E-06	12.14	36.92
		Emory	-24.28	9.17	0.027	-46.43	-2.13
	Stavanger	Nottingham	-24.53	5.16	9.43E-06	-36.92	-12.14
		Emory	-48.81	9.25	1.43E-06	-71.15	-26.47
	Emory	Nottingham	24.28	9.17	0.027	2.13	46.43
		Norway	48.81	9.25	1.43E-06	26.47	71.15
HER3-EGFR	Nottingham	Norway	117.63	8.81	0.00E+00	96.46	138.79
		Emory	101.83	10.97	0.00E+00	75.42	128.23
	Stavanger	Nottingham	-117.63	8.81	0.00E+00	-138.79	-96.46
		Emory	-15.80	11.12	0.40	-42.61	11.00
	Emory	Nottingham	-101.83	10.97	0.00E+00	-128.23	-75.42
		Norway	15.80	11.12	0.40	-11.00	42.61

Table S4. Multivariate categorical regression analysis of HER3, EGFR, and HER3-EGFR protein expression. SE=standard error of the mean; zero=zero-order; import=importance

		Coefficients				Correlations and Tolerance					
		Standardized Coefficients				Correlations				Tolerance	
Dependent variable	Covariate	Beta	Bootstrap (1000) SE	F	p-value	Zero	Partial	Part	Import	After Transform	Before Transform
HER3	Age	0.06	0.04	2.51	0.11	-0.09	0.07	0.06	-0.01	0.78	0.79
	Chemo	0.08	0.05	3.02	0.08	-0.23	0.08	0.06	-0.04	0.61	0.66
	Grade	0.06	0.04	2.81	0.06	0.10	0.08	0.06	0.02	0.98	0.96
	Stage	-0.03	0.03	0.85	0.36	0.03	-0.04	-0.03	0.00	0.91	0.94
	Cohort	0.68	0.03	435.35	0.00E+00	0.64	0.59	0.56	1.04	0.68	0.73
EGFR	Age	0.09	0.05	3.02	0.08	0.09	0.08	0.08	0.12	0.88	0.79
	Chemo	-0.07	0.05	2.21	0.14	-0.02	-0.07	-0.07	0.02	0.88	0.66
	Grade	-0.04	0.04	0.87	0.35	-0.04	-0.04	-0.04	0.02	0.97	0.96
	Stage	0.05	0.04	1.43	0.24	0.06	0.05	0.05	0.05	0.98	0.94
	Cohort	-0.23	0.04	28.90	1.65E-12	-0.23	-0.23	-0.23	0.79	0.98	0.73
HER3-EGFR	Age	0.10	0.04	5.55	0.02	-0.01	0.10	0.09	0.00	0.79	0.79
	Chemo	0.10	0.05	3.75	0.05	-0.17	0.09	0.08	-0.07	0.64	0.66
	Grade	0.04	0.03	1.42	0.24	0.03	0.04	0.04	0.01	0.99	0.96
	Stage	0.04	0.04	0.99	0.32	0.03	0.04	0.04	0.01	0.99	0.94
	Cohort	0.53	0.04	150.73	0.00E+00	0.46	0.45	0.44	1.07	0.69	0.73

Table S5. Differential transcript expression by HER3-EGFR group. *See attached file.*

Table S6. Differential transcript expression by HER3-EGFR group after adjusting for age at diagnosis and AJCC stage. *See attached file.*
